# Vasoactive intestinal peptide regulates ileal goblet cell production in mice

**DOI:** 10.14814/phy2.14363

**Published:** 2020-02-05

**Authors:** Luke A. Schwerdtfeger, Stuart A. Tobet

**Affiliations:** ^1^ Department of Biomedical Sciences Colorado State University Fort Collins CO USA; ^2^ School of Biomedical Engineering Colorado State University Fort Collins CO USA

**Keywords:** goblet cell, intestine, mucosa, organotypic, VIP

## Abstract

Innervation of the intestinal mucosa has gained more attention with demonstrations of tuft and enteroendocrine cell innervation. However, the role(s) these fibers play in maintaining the epithelial and mucus barriers are still poorly understood. This study therefore examines the proximity of mouse ileal goblet cells to neuronal fibers, and the regulation of goblet cell production by vasoactive intestinal peptide (VIP). An organotypic intestinal slice model that maintains the cellular diversity of the intestinal wall ex vivo was used. An ex vivo copper‐free click‐reaction to label glycosaminoglycans was used to identify goblet cells. Pharmacological treatment of slices was used to assess the influence of VIP receptor antagonism on goblet cell production and neuronal fiber proximity. Goblet cells were counted and shown to have at least one peripherin immunoreactive fiber within 3 µm of the cell, 51% of the time. Treatment with a VIP receptor type I and II antagonist (VPACa) resulted in an increase in the percentage of goblet cells with peripherin fibers. Pharmacological treatments altered goblet cell counts in intestinal crypts and villi, with tetrodotoxin and VPACa substantially decreasing goblet cell counts. When cultured with 5‐Ethynyl‐2’‐deoxyuridine (EdU) as an indicator of cell proliferation, colocalization of labeled goblet cells and EdU in ileal crypts was decreased by 77% when treated with VPACa. This study demonstrates a close relationship of intestinal goblet cells to neuronal fibers. By using organotypic slices from mouse ileum, vasoactive intestinal peptide receptor regulation of gut wall goblet cell production was revealed.

## Graphical abstract

A close relationship between neuronal fibers and enteric goblet cells was demonstrated, with more neuronal fibers being in close proximity to goblet cells in vasoactive intestinal peptide (VIP) receptor antagonized ileum slices. Further, antagonizing VIP receptors ex vivo was shown to regulate goblet cell production in the ileal crypt.

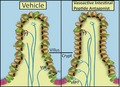

## 1 INTRODUCTION

Gut luminal microbiota are separated from the small intestinal epithelial barrier by an nonadherent mucus layer in the small intestine (Kaelberer et al., 2018). Goblet cells in the intestinal wall secrete large quantities of mucus, composed of mucins – principally Muc2 in intestine – which are comprised of a protein core, connected to chains of O‐linked glycans, usually in the form of glucosamino‐ or glycosaminoglycans (Holmen Larsson, Thomsson, Rodriguez‐Pineiro, Karlsson, & Hansson, 2013). How the gut mucus layer and epithelial barrier are regulated in health and disease is poorly understood. This is partially due to investigations using monolayer epithelial culture models that until recently did not contain a mucus layer (Wang, Kim, Sims, & Allbritton, 2019). While this is an advance, epithelial culture systems miss the cellular diversity of the gut wall, a critical aspect of in vivo physiologic function (McLean, Schwerdtfeger, Tobet, & Henry, 2018; Schwerdtfeger & Tobet, 2019). Understanding intestinal wall function ultimately requires parsing the interactions of the diverse cellular elements. The current study addresses the impact of neural regulation on gut wall goblet cells in an organotypic model of gut physiology that maintains numerous cell types ex vivo, including goblet cells and neurons.

There have been several recent demonstrations of neural influence(s) on gut epithelial components, principally on secretory epithelial cell types (Walsh & Zemper, 2019). One line of study focused on glial – enteroendocrine cell (EEC) interactions (Bohorquez et al., 2014) and more recently, direct innervation of EECs via vagal afferents (Kaelberer et al., 2018). Another line of study focused on tuft cells and show close proximity to neuronal fibers, with more connections observed in the proximal small intestine (Cheng, Voss, & Ekblad, 2018). There are peptidergic neuronal fibers throughout the intestinal mucosa (Goyal & Hirano, 1996) in close proximity to the apical enterocytes in the subepithelial plexus (Furness & Costa, 1987; Keast, Furness, & Costa, 1985). Whether, and which, peptides/factors regulate goblet cell function is unclear. Corticotropin‐releasing hormone (CRH) has been shown to stimulate mucus secretion in the colon of rats (Pothoulakis, Castagliuolo, & Leeman, 1998), and blocking neuronal firing with tetrodotoxin has been shown to inhibit goblet cell secretion normally evoked by electrical field stimulation (Phillips, Phillips, & Neutra, 1984). Vasoactive intestinal polypeptide (VIP) has been shown to cause goblet cell secretion (Dartt, Kessler, Chung, & Zieske, 1996; Kirkegaard et al., 1981), but other reports have not found influences of VIP on intestinal goblet cell mucus secretion (Halm & Halm, 2000; Neutra, O'Malley, & Specian, 1982). Given the critical role goblet cells play in maintaining the gut mucus layer ( Allaire et al., 2018) and in direct interactions with microbiota (Birchenough, Nystrom, Johansson, & Hansson, 2016; Jakobsson et al., 2015; Knoop, McDonald, McCrate, McDole, & Newberry, 2015), this study was conducted to further delineate the peptide regulation of goblet cell function.

VIP is a 28‐amino‐acid peptide secreted by enteric neurons (Furness & Costa, 1979; Sikora, Buchan, Levy, Mcintosh, & Brown, 1984), and has known to play a role in gut smooth muscle contractility/relaxation (Katsoulis, Clemens, Schworer, Creutzfeldt, & Schmidt, 1993) and ion secretion (Cooke, 1994). While VIP shares high sequence homology with pituitary adenylate cyclase‐activating polypeptide (PACAP), both peptides bind the same receptors, VPAC1 and VPAC2, however at slightly different affinities (Vaudry et al., 2009). Both receptors can be antagonized by [D‐p‐Cl‐Phe^6^,Leu^17^]‐VIP (Pandol, Dharmsathaphorn, Schoeffield, Vale, & Rivier, 1986) which will be referred to as “VPACa”. Given the large distribution of VIP receptors throughout the gut wall (Jayawardena et al., 2017), it is reasonable to consider the potential of neuronal regulation in goblet cell quantities, production of mucus, and/or secretion, at baseline and potentially in response to bacterial infiltration.

This study uses organotypic intestinal slices (Schwerdtfeger, Nealon, Ryan, & Tobet, 2019; Schwerdtfeger, Ryan, & Tobet, 2016) as a platform for investigating neural – goblet cell interactions in mouse ileum ex vivo. This intestinal slice method allowed for use of three pharmacological tools for altering goblet cell function ex vivo in a cellularly heterogenous tissue. Lipopolysaccharide (LPS) causes intestinal goblet cells to secrete mucus in mouse colon, but not ileum ( Birchenough et al., 2016). The sodium ion channel blocker tetrodotoxin (TTX), which blocks large amounts of enteric neuronal signaling (Osorio, Korogod, & Delmas, 2014), and finally VPACa, an antagonist for both VIP receptors (Pandol et al., 1986). These ex vivo pharmacological treatments were coupled with molecular visualization tools to reveal anatomical bases for neuronal fiber signaling with ileal goblet cells. Further, this study suggests a specific role for neuronal fibers containing VIP to play in regulating intestinal goblet cell production.

## 2 MATERIALS AND METHODS

### 2.1 Animals

Male and female adult mice aged between 8‐ and16‐week‐old, of the C57BL/6 background were used for all experiments. Mice were housed at Colorado State University, under the care of Laboratory Animal Resources, and kept in cages with aspen bedding (autoclaved Sani‐chips; Harlan Teklad, Madison, WI). Mice were housed under a 14:10‐hr light‐dark cycle, with ad libitum access to water and food (no. 8,649; Harlan Teklad). Intestinal slices were generated from a transgenic strain where animals expressed yellow fluorescent protein (YFP) driven by a neuronally selective Thy‐1 promoter (Feng et al., 2000) (Thy‐1 YFP). Animal studies were approved by the Colorado State University IACUC under protocol #17‐7270a. Intestines from at least three animals were used for all experiments and matched by sex where possible.

### 2.2 Organotypic slice preparation

Preparation of intestinal slices was similar to that previously described (Schwerdtfeger et al., 2016). Briefly, mice were deeply anesthetized with isoflurane and subsequently killed via decapitation, to ensure severing of vagal fibers. The entire small intestine was removed from the pylorus‐duodenal junction, to the ileo‐cecal junction. The tissue was immediately placed into 4°C 1X Krebs buffer and the ileum was separated from the remainder of the intestine‐based off tissue anatomy. The remnant mesenteric fat and connective tissue was dissected away, and the tissue was cut into pieces roughly 2–4‐mm in length. The tissue was submerged in low‐melting point, 8% agarose (Gold Biotechnology), 5 min in a room temperature shaker, and 2 min in 4°C to ensure gelation. Slices of 250 µm thick were cut on a vibrating microtome (VT1000S; Leica Microsystems, Wetzlar, Germany) and collected into ice cold 1X Krebs buffer, before transfer into a 60 mm plastic‐bottom dish (Corning, Corning, NY) containing 5 ml of Hibernate Media (Life Technologies). Slices spent 15 min at 4°C in Hibernate media prior to being transferred into 5 ml of CTS Neurobasal‐A Media (ANB; Life Technologies) with 5% B‐27 supplement (B‐27; Life Technologies) where they spent 35 min at 37°C. Samples were plated on 35 mm plastic bottom dishes (MatTek, Ashland, MA) with excess media being siphoned from the dish, and incubated at 37°C for 10 min. Next, slices were covered by a thin layer of collagen solution [vol/vol: 10.4% 10X MEM (Minimal Essential Medium, Sigma‐Aldrich), 4.2% sodium bicarbonate, and 83.5% collagen (PureCol; Inamed)], which was allowed to polymerize for 15 min prior to final addition of 1 ml of ANB + B‐27. Tissue was left in a 37°C, 5% CO_2_, and 1% O_2_ incubator until further experiments were performed.

### 2.3 Glycosaminoglycan visualization and drug dosing

Slices were created as above and cultured in ANB with B‐27 for 24 hr prior to the addition of an azido‐modified galactosamine, Tetraacetylated N‐Azidoacetylgalactosamine (GalNAz; 12.5 µM; Fisher Scientific). GalNAz was allowed to incubate in the slice dishes for 24 hr prior to development. Concurrently with GalNAz treatment, slices were dosed with one of four compounds at 24 hr ex vivo: vehicle (10 µl Milli‐Q Water), TLR grade lipopolysaccharide derived from *E. coli* Serotype EH100 (10 µg/ml; Enzo Life Sciences, Inc. Farmingdale, NY), the sodium ion channel blocker tetrodotoxin (10 µM; Abcam, Cambridge, MA) or the vasoactive intestinal peptide receptor antagonist [D‐p‐Cl‐Phe^6^,Leu^17^]‐VIP (10 µM, Bio‐Techne Corporation, Minneapolis, MN). After 24 hr of incubation, the fluorophore‐tagged alkyne, Dibenzocyclooctyne‐Cy3 (DBCO‐Cy3; 2 µM; Sigma‐Aldrich, St. Louis, MO) was added to visualize GalNAz. This copper‐free click reaction was allowed to proceed in the dark for 15 min at 37°C, 5% CO_2_, 1% O_2_. Finally, the culture supernatant was removed, and slices were fixed in 4% formaldehyde prior to resectioning.

### 2.4 Resectioning of slices

**Figure 1:**
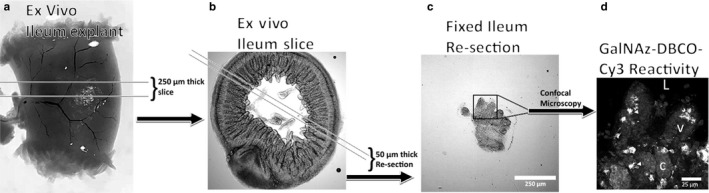
Schematic representation of culture protocol path from a ~ 2cm long ileum explant (a) to a 250 µm thick ex vivo ileum slice (b) to a 50 µm thick resectioned piece of fixed ileum (c), and a representative confocal photomicrograph of GalNAz‐DBCO‐Cy3 reactivity (d). In D, arrow heads point to stereotypic GalNAz‐DBCO‐Cy3 labeled cells, and ‘L’ represents the lumen, ‘v’ a villus, and ‘c’ a crypt. Scale bars are 250 µm in (c), and 25 µm in (d)

After 48h of culture, ileum slices were fixed for 10 min in 4% formaldehyde. Tissue was then placed in a 4% agarose solution (w/v; Fisher Scientific) and subsequently put in a 4°C fridge for 4 min to ensure agarose gelation. Ileum slices were then sectioned on a vibrating microtome (VT1000S; Leica Microsystems) at 50 µm thick (Figure 1c) before being processed for immunohistochemistry.

### 2.5 Immunohistochemistry

After resectioning, 50 µm sections were washed in PBS for at least 10 min prior to receiving 0.1M glycine made in 0.05M PBS for 30 min. The tissue was subsequently washed three times in PBS for 5 min each wash. Next, sections received 0.5% sodium borohydride in PBS for 15 min. Sections were then washed twice for 5 min in PBS before blocking in 5% NGS, 0.5% Tx, and 1% H_2_O_2_ in PBS for 30 min. After blocking, sections received one of three primary antisera for two days: a monoclonal anti‐peripherin (1:300; Chemicon International, Temecula, CA), a polyclonal anti‐VIP (1:8,000; Immunostar, Inc. Hudson, WI), or a polyclonal anti‐MUC2 (3 µg/ml; Novus Biologicals). After primary sections were washed with 1% NGS in PBS four times for 15 min each wash. Next, secondary antibody was added for 2 hr at room temperature and consisted of 1% NGS and 0.5% Tx in PBS with a biotinylated goat anti‐rabbit secondary antibody (1:2,500; Jackson Immunoresearch Inc. West Grove, PA). Secondary antibody was washed out with four 15 min washes composed of 0.02% Tx in PBS. Sections were next incubated with an Alexa Fluor 488 conjugated to streptavidin (1:500; Invitrogen) in 0.32% Tx in PBS for 1 hr. Finally, sections received three PBS washes prior to mounting and imaging.

### 2.6 Tissue imaging and analysis

Slices and resectioned tissue were imaged on either a Nikon TE2000‐U inverted microscope (10X Plan‐Fluor and 20X Plan‐Apo objectives) with a UniBlitz shutter system (Vincent Associates, Rochester, NY) and an Orca‐flash 4.0 LT camera (Hamamatsu, Hamamatsu City, Shizuoka Prefecture, Japan), or a Zeiss LSM 880 confocal microscope with an Axiocam 503 mono camera (Carl Zeiss, Inc., Thornton, NY). Data in GalNAz‐DBCO‐Cy3 fluorescent cell counting and the EdU/ GalNAz colocalization experiments were gathered via confocal Z‐stack with 30 planes, 1 µm apart being captured through the center of the tissue. A max intensity Z‐projection was performed using FIJI (ImageJ, v1.0; NIH) and cells were manually counted by a researcher blinded to treatment. Data for GalNAz‐DBCO‐Cy3 fluorescent cell proximity to peripherin immunoreactive fibers was performed by a researcher blinded to treatment who randomly sampled 3‐ Z‐planes throughout the tissue section based on GalNAz‐DBCO‐Cy3 fluorescence only. Analysis was performed in FIJI using a 3 µm dilation around all GalNAz‐DBCO‐Cy3 fluorescent cells before the analyzing particles tool to quantify peripherin immunoreactive fibers within the 3 µm dilations.

### 2.7 Statistics

All statistical analysis was performed using Prism 8 (Graphpad). For all GalNAz cell count analysis and peripherin‐GalNAz analysis, a two‐way ANOVA was performed by treatment and region. A Sidak's multiple comparisons post hoc test was performed for comparison of individual group means. For the GalNAz – EdU colocalization experiment, data was analyzed using a one‐way ANOVA by treatment, with a Tukey's multiple comparisons post hoc test. All data are presented as means ± standard error of the mean (*SEM*).

## 3 RESULTS

### 3.1 Intestinal slices maintain goblet cells ex vivo

Organotypic intestinal slices from mouse ileum (Figure 1a,b) maintained glycosaminoglycan producing goblet cells ex vivo for at least 48 hr. Labeling of glycosaminoglycans was accomplished using a copper free azide‐alkyne cycloaddition (CFAAC; Figure S1) click reaction. Incorporation of Tetraacetylated N‐Azidoacetylgalactosamine (GalNAz) into goblet cell glycosaminoglycans was visualized with dibenzocyclooctyne‐Cy3 (DBCO‐Cy3) and thereby labeled glycosaminoglycan producing goblet cells ex vivo (Figure 1c,d). For preliminary experiments, slices of mouse colon from 4 animals per treatment were cultured in either atmospheric oxygen conditions at 5,000 feet above sea level in Colorado (~17% = 100 mmHg) or low oxygen (1% = 5.9 mmHg) incubators. The percentage of area labeled in regions of interest drawn around colonic mucosa with GalNAz‐DBCO‐Cy3 fluorescence was measured. GalNAz‐DBCO‐Cy3 label was 2‐fold higher in 1% cultured slices compared to 17% oxygen cultured tissue, with slices cultured in 1% oxygen showing a mean of 20.16 ± 2.3 percentage area labeled, while 17% oxygen slices had 10.55 ± 1.9 percent area labeled ([*t* = 3.23, *df* = 33] *p* < .01). Area analyzed was similar for both conditions and incorporated dozens of crypts per treatment. Mean area analyzed was 409.8 ± 43.3 mm^2^ for 17% oxygen slices, and 306.5 ± 56.7 mm^2^ for 1% oxygen slices ([*t* = 1.436, *df* = 33] *p* = .16). All subsequent experiments were conducted in the low oxygen environment. Organotypic intestinal slices from mouse ileum housed GalNAz‐DBCO‐Cy3 labeled cells along the length of the crypt‐villus axis. Post hoc immunohistochemistry showed regular colocalization of MUC2 immunoreactivity with GalNAz‐DBCO‐Cy3 fluorescence (Figure S2a,b). Across all treatments in mouse ileal slices, GalNAz‐DBCO‐Cy3 fluorescent cells were colocalized with immunoreactive MUC2 62% of the time.

### 3.2 Neural fibers densely innervate ileal mucosa

**Figure 2:**
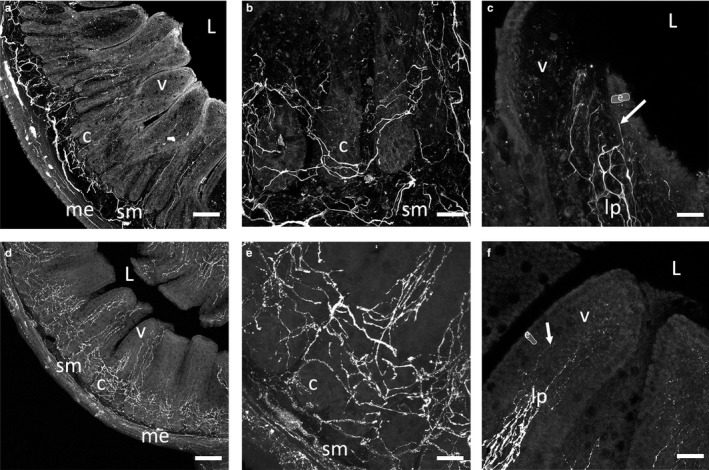
Representative z‐projected confocal images of peripherin (a–c) and VIP (d–f) immunoreactivity in 50 µm sections of mouse ileum. Panels (a) and (d) show patterns of fiber distribution throughout the mucosa. Panels (b) and (e) show dense ileal crypt innervation patterns, and panels (c) and (f) show neuronal fibers in the villi, with arrows pointing to fibers of the subepithelial plexus running beneath the apical‐most epithelial layer. Outlined and highlighted areas labeled with ‘e’ in panels (c) and (f) represent apical enterocytes. High magnification images in panels (b) and (c) are not directly taken from the same section as the low‐magnification image in panel (a). In all panels, ‘L’ represents the lumen, ‘v’ a villus, ‘c’ a crypt, ‘sm’ submucosa, ‘me’ muscularis externa, and ‘lp’ lamina propria. Scale bars 100 µm in (a) and (d), and 25 µm in b,c,e,f

An extensive network of enteric neuronal fibers infiltrated the gut mucosa. Fibers containing immunoreactive peripherin densely wrapped around intestinal crypts weaving throughout the lamina propria toward the intestinal lumen (Figure 2a,b). In these same sections, peripherin immunoreactive fibers were projected to the apical most enterocytes (outlined and highlighted in Figure 2c and f) that form the gut epithelial barrier (arrow, Figure 2c). VIP – immunoreactive fibers were also observed densely wrapping intestinal crypts (Figure 2d,e) and throughout the villus lamina propria and extended to the villus apex (arrows, Figure 2f). These fibers were detected projecting directly into the apical most enterocytes in the ileal villi, as with peripherin fibers.

### 3.3 Peripherin fibers project to ileal goblet cells

**Figure 3:**
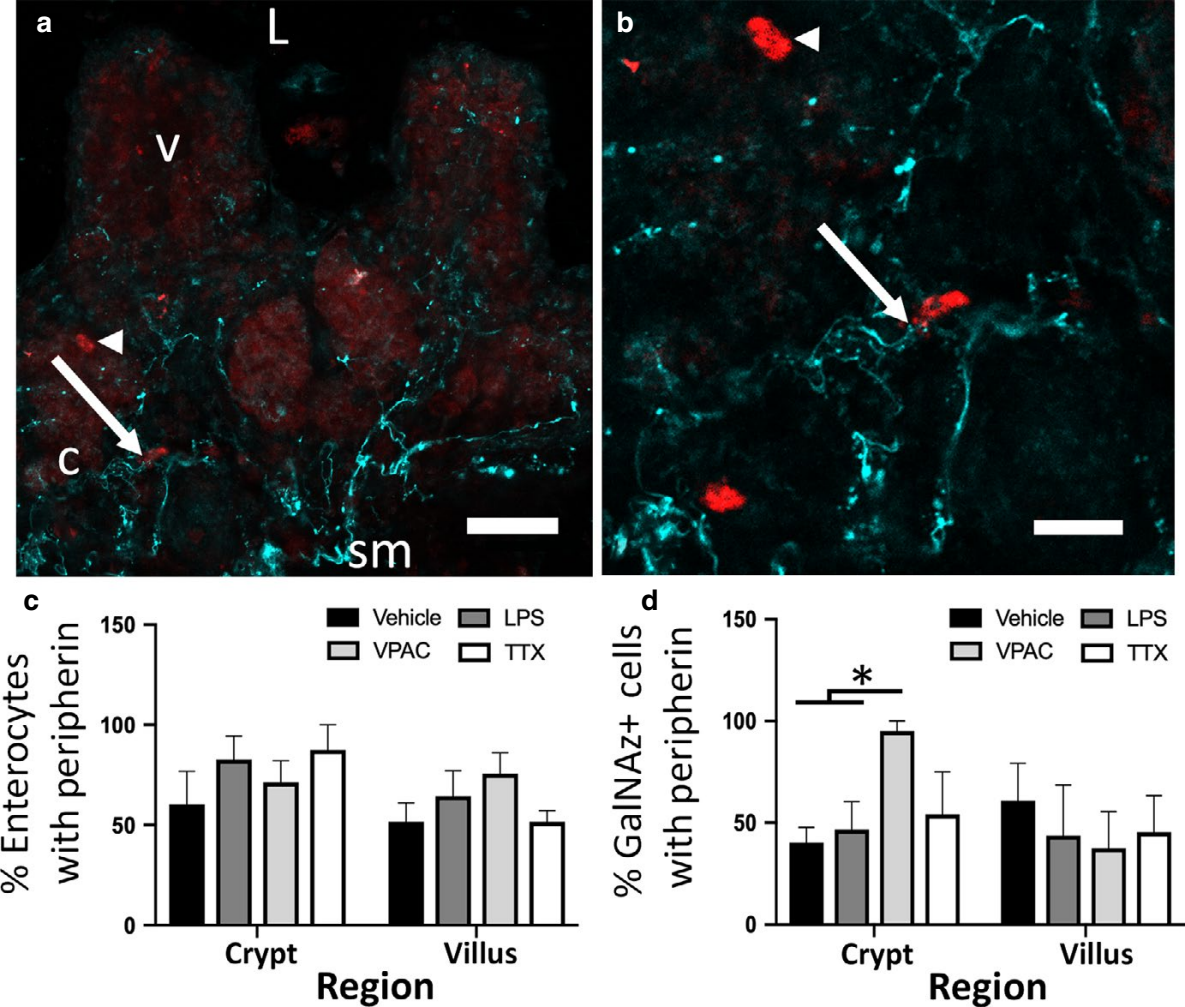
Percentage of GalNAz + cells with peripherin fibers within 3µm is increased in VPACa treatment in ileal crypts. Representative photomicrograph (a) shows GalNAz‐DBCO‐Cy3 reactive cells in red (arrow and arrow head) with peripherin‐immunoreactive fibers in cyan throughout the mucosa. (a’) is a magnified view of (a) showing numerous fibers projecting toward a GalNAz‐DBCO‐Cy3 reactive cell (arrow), and a second cell without any fibers (arrow head). (b) quantification of the percentage of non‐GalNAz reactive enterocytes peripherin fibers. (c) quantification of the percentage of GalNAz‐DBCO‐Cy3 reactive cells with peripherin fibers. ‘L’ represents the lumen, ‘v’ a villus, ‘c’ a crypt. The * in panel B denotes *p* < .05. Scale bars are 25 µm in (a) and 10 µm in (a’). Data from *n* = 4 animals (2 males, 2 females). Values are means ± *SEM*

Neuronal fibers were in close proximity (within 3 µm) to GalNAz‐DBCO‐Cy3 reactive goblet cells throughout the mucosa (Fiber 3A‐A’; arrow). Neuronal fibers containing immunoreactive peripherin were denser in the submucosal and crypt regions of ileal tissue (Figure 3a) than in the villi. This was consistent with both peripherin‐ and VIP‐ immunoreactivity patterns in fixed 50 µm sections of ileum (Figure 2a–c). Numerous fibers were found in close proximity to GalNAz‐DBCO‐Cy3 fluorescent goblet cells (e.g., arrow in Figure 3a–a’). There were also many goblet cells that did not have closely apposed peripherin fibers (arrow head in Figure 3a–a’). In addition, when looking at enterocytes directly adjacent to GalNAz labeled cells, there was no change in percentage of these enterocytes with proximal peripherin fibers, regardless of region ([*F*(1,24) = 3.18]; *p* = .09) or treatment (Figure 3b; [*F*(3,24) = 1.02]; *p* = .4). When analyzing GalNAz‐DBCO‐Cy3 reactive cells, vehicle‐treated slices had 53.9 ± 9.2 percent of goblet cells with at least one peripherin fiber. LPS‐treated slices showed 44.7 ± 12.4 percent with a fiber, while VPACa and TTX had 56.7 ± 11.7 and 48.4 ± 10.4 percent of cells with a fiber, respectively. No differences were observed across these treatments ([*F*(3,44) = 0.24]; *p* = .86). When separated by anatomic region, no differences were observed in the percentage of GalNAz‐DBCO‐Cy3 reactive cells with closely apposed peripherin fibers across all treatments ([*F*(3,24) = 0.59]; *p* = .62) or by region (i.e. crypt vs. villus; [*F*(1,24) = 1.03]; *p* = .32) except for slices treated with VPACa. Slices treated with VPACa for 24 hr showed an increase in the percentage of GalNAz‐DBCO‐Cy3 fluorescent cells with peripherin fibers in the crypt (95 ± 5 percent) compared to vehicle (40.3 ± 7.5) and LPS (46.7 ± 13.7; Figure 3c [*F*(3,11) = 6.07]; *p* = .01).

### 3.4 VPAC receptors regulate goblet cell count and production

**Figure 4:**
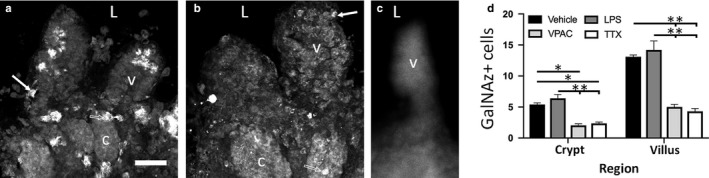
Dosing with VPACa or TTX substantially decreases the quantity of GalNAz + cells per crypt, and per villus compared to vehicle and/or LPS. (a) a representative photomicrograph of a vehicle‐treated slice showing large quantities of GalNAz‐DBCO‐Cy3 reactive cells in both the crypts ‘c’ and the villi ‘v’. (b) a representative photomicrograph of DBCO‐Cy3 reactive cells in a VPACa‐treated slice. Arrows in both A and B point to a stereotypic DBCO‐Cy3 labeled cell in the villus, while hollow arrows point to a stereotypic cell in the crypt. (c) Representative negative control image showing a lack of clearly labeled goblet cells. (d) quantification of the GalNAz + cell counts throughout the crypt and villi. ‘L’ represents the lumen. In panel (c), * denotes *p* < .05, and ** denotes *p* < .01. Scale bars in (a) – (c) are 25 µm. Data from *n* = 6 animals (3 males, 3 females). Values are means ± *SEM*

**Figure 5:**
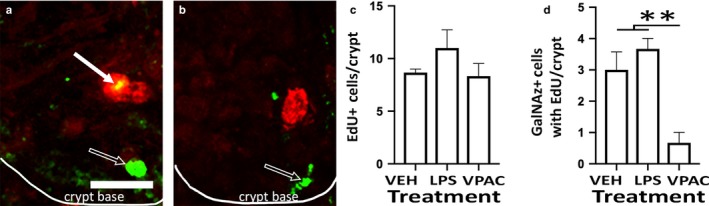
Dosing with VPACa decreased the number of GalNAz + cells with EdU colocalization. (a) shows the base of a single crypt in a vehicle‐treated slice, with a GalNAz‐DBCO‐Cy3 reactive cell (red) colocalized with EdU (green). In A there is an EdU reactive cell (hollow arrow) and a colocalized cell (arrow). (b) shows the base of a single crypt in a VPACa‐treated slice, and an EdU reactive cell (hollow arrow), and a GalNAz‐DBCO‐Cy3 reactive cell (red) without any EdU colocalization are visible. White line denotes the crypt base in (a–b). (c) quantification of total EdU cell counts per crypt. (d) quantification of GalNAz – EdU colocalization. Scale bar in (a) is 5 µm. Scale in (b) is the same as (a). Data from *n* = 3 animals (2 males, 1 female). Values are means ± *SEM*

GalNAz‐DBCO‐Cy3 fluorescent goblet cell count and proliferation were altered by ex vivo pharmacological treatment with VPACa. There was a 50% decrease in GalNAz + cell counts in the crypts and villi compared to vehicle and/or LPS (Figure 4) that can be seen in a representative image of a vehicle‐treated slice (Figure 4a) compared to a VPACa‐treated slice (Figure 4b). When separated by region, more GalNAz‐DBCO‐Cy3 reactive cells were observed in the ileal villi compared to crypts across treatments (Figure 4a‐d; [*F*(3,32) = 131.4]; *p* < .0001). This effect was also observed between vehicle and VPACa, as well as vehicle and TTX (Figure 4d; [*F*(3,32) = 68.9]; *p* < .0001). Negative controls were performed for the CFAAC reaction, showing no fluorescently labeled goblet cells when mouse ileal slices were cultured with DBCO‐Cy3 alone, without GalNAz (Figure 4c). When vehicle (Figure 5a) and VPACa (Figure 5b) treated slices were given the thymidine analog 5‐Ethynyl‐2’‐deoxyuridine (EdU) ex vivo, incorporation of EdU was detected in 9.3 ± 1.09 cells/ crypt across all treatments. No differences were observed in EdU cell counts per crypt between treatments (Figure 5c). Slices treated with EdU and GalNAz‐DBCO‐Cy3 ex vivo showed colocalization in ~30% of all EdU cells in ileal crypts in vehicle‐treated slices (Figure 5a,d). When treated with LPS, there was no difference compared to control. However, treatment with VPACa (Figure 5b) resulted in a 77% decrease in the number of GalNAz‐DBCO‐Cy3 reactive cells that were colocalized with EdU (Figure 5d; [*F*(2,6) = 13.4]; *p* < .01).

## 4 DISCUSSION

The intestinal wall functions as an ensemble of cellular constituents that provide for nutrient absorption on one hand and protection from the outside world on the other. From the luminal space to the outside of the wall, the constituents include microbiota, mucus, and then epithelial, immune, neural, and muscle cells. Communication among these elements is extensive, but poorly defined in many places. This study provides an anatomical basis for neural signaling to enteric goblet cells of the epithelial layer and shows the selective impact of VIP receptors on goblet cell production. There have been demonstrations of neural – epithelial signaling in the intestine via enteroendocrine cells (Bohorquez et al., 2014, 2015), and cholinergic regulation of goblet cells in the eye conjunctiva (Garcia‐Posadas et al., 2016). Peptidergic regulation of goblet cells in the intestine has been suggested, but potential sources have been vague. The close proximity of enteric neuronal fibers to a subset of goblet cells labeled for live mucus synthesis in this study provides an anatomical linkage for potential functional signaling between these cells. Goblet cells with live glycosaminoglycan labeling in the crypts were more likely to be in close proximity to peripherin immunoreactive neuronal fibers when slices were treated ex vivo with a VIP antagonist (VPACa) compared to control, an inhibitor of synaptic signaling through sodium channels (tetrodotoxin), or an immune system stimulant (LPS). Thus, VIP of local origin may be a key modulator of goblet cell function in the intestinal wall.

This study adapted a powerful technique to label goblet cells and mucus in vivo (Johansson, Larsson, and Hansson (2011)) to label ex vivo and ultimately will make it possible to examine live. Copper‐catalyzed azide‐alkyne additions have been used but are traditionally thought to be cytotoxic to living cells and tissues (Baskin et al., 2007). Copper‐free azide‐alkyne cycloadditions (CFAACs) have gained some prominence due to their biocompatibility and have been used to label intestinal goblet cells in fixed tissue (Schneider, Pelaseyed, Svensson, & Johansson, 2018). CFAACs have not previously been used ex vivo to label goblet cells in cellularly heterogenous tissues. This study verified that a CFAAC method labeled live glycosaminoglycan producing intestinal goblet cells ex vivo. GalNAz incorporation and subsequent label via CFAAC with a fluorophore had a consistent colocalization with Muc2‐immunoreactivity in ~62% of cells. This colocalization matched a previous study that labeled mouse colonic goblet cells in vivo with GalNAz and subsequently labeled with Muc2 via immunohistochemistry (Johansson et al., 2011). These data indicate that the percent incorporation of GalNAz into a Muc‐2 immunoreactive goblet cells in ileum is similar ex vivo as in vivo removing the concern of tissue toxicity due to copper while retaining the labeling efficiency. Whether the percent of goblet cells labeled for glycosaminoglycan synthesis being less than the total indicates unique attributes of the cells such as synthesis of sugars other than galactosamines, or is due to limitations of the technique as suggested previously (Johansson, 2012) remains to be determined.

The presence of a subepithelial plexus of neural fibers in intestinal villi was first observed by Ramon y Cajal, among others, in the late 1800s (Cajal, 1894; Furness & Costa, 1987). However, in‐depth phenotyping of these fibers along the length of the gastrointestinal tract is still incomplete. Neuronal fibers carrying immunoreactive VIP have been shown to travel in close proximity to enterocytes in the intestinal crypts (Wu et al., 2015), however, the role these fibers play in regulating goblet cells (production and/or function) is unclear. In the present study, VIP receptor antagonism affected neuronal fiber localization close to goblet cells in the ileal crypts, but not villi. Therefore, VIP’s role may be spatially limited, and other transmitters may be involved in goblet cell signaling as they ascend the length of the villi. There are a large number of neuronally secreted peptides in the intestine that may play roles in modulating goblet cell secretion (Furness & Costa, 1987), including corticotrophin‐releasing hormone (Castagliuolo et al., 1996), substance P (Wagner et al., 1995), somatostatin (Wagner et al., 1995), and calcitonin gene‐related peptide (Plaisancie et al., 1998), among others. Additionally, bi‐directional neuroimmune signaling between enteric neuronal fibers and mast cells (Buhner et al., 2017) can regulate mucus secretion (Castagliuolo et al., 1996) among a host of other cellular responses (Bednarska et al., 2017). Therefore, VIP produced in neurons is not the only pathway for peptidergic regulation of goblet cell functions, and further fleshing out of the molecular signaling mechanisms between enteric neuronal projections, mast cells, and goblet cells will be important.

Enteric neurons express a large variety of receptors, produce numerous different peptides and transmitters, and vary in their excitatory state dependent upon region (Nurgali, 2009). TTX blocks voltage‐gated sodium channels, of which there are numerous types in the intestine, with differential expression based on neuron type (Bartoo, Sprunger, & Schneider, 2005). Inhibition of sodium channels via TTX dosing served as a more global, nonspecific, neuronal inhibition for pharmacological studies in which the specific receptor antagonist, VPACa, was used. Observations in this study showed similar influences of TTX and VPACa on the counts of goblet cells incorporating GalNaz ex vivo in both crypts and villi. This points toward a neural (TTX) influence on goblet cells and a potentially more specific pathway via VIP receptors (VPACa). Further work is needed to explicitly define the peptide receptor expression on enteric goblet cells, with a focus on crypt and villi cell populations as a distinct variable.

Goblet cell function in the intestinal tract is likely regulated by neural factors, however, there is a large intestinal immune component that holds influence over epithelial function. The pathways involved in goblet cell signaling with neurons, immune cells, or both, remain poorly understood. Treatment with the bacterial cell wall component LPS has been shown to increase mast cell activation (Cho, Park, Kim, Choo, & Lee, 2018). While mast cells have been observed to be involved in enteric neuro‐immune signaling (Buhner et al., 2017), and are known to have VIP‐receptors (Keita et al., 2013), there was no direct influence of LPS on the counts of goblet cells incorporating GalNaz ex vivo in any of the present experiments. This suggests that there may not be a direct immune signaling altering glycosaminoglycan synthesis in goblet cells or their proximity to neuronal fibers. Given the influence of VPACa dosing on neuronal fiber localization and goblet cell counts, VIP may be a key regulator of goblet cells in the ileal mucosa. However, given the likely role of neural – immune signaling in host‐pathogen interactions (Sharkey, Beck, & McKay, 2018), further work is needed to flesh out more obscure influences of the immune system on enteric goblet cell regulation.

The results of this study are consistent with the hypothesis that epithelial cell functions (e.g., goblet cell production) in the small intestine are modulated by peptides normally produced in neurons, such as VIP. There is a dense network of neuronal fibers wrapping around intestinal crypts (Furness & Costa, 1987). The mechanisms of signaling between neural fibers and crypt epithelia remains unclear. Incorporation of the thymidine analog EdU into DNA has previously been used to quantify the rate of epithelial cell production in mouse small intestine both in vivo (Krndija et al., (2019)) and ex vivo ( Schwerdtfeger et al., 2016). In this study, the number of glycan synthesizing goblet cells that were also undergoing DNA synthesis as marked by EdU was substantially decreased in slices treated with the VIP receptor antagonist VPACa. This indicates potential ongoing VIP regulation of goblet cell production. Further investigation is required to determine whether this was a direct impact on goblet cell proliferation from a progenitor, or a later influence on differentiation into a goblet cell fate.

In conclusion, this study provides an anatomical foundation for neural influences on goblet cell function in the mouse ileum. Further, these interactions are seemingly important for regulation of the extent of the proximity of neural fibers to goblet cells, and the production of new goblet cells in the ileal crypt. The experiments in this study lay a foundation for analyzing goblet cell‐neuronal interactions in the mouse ileum. Future investigation will be needed to tease apart the role of other neuropeptides in mediating neural interactions with goblet cells along the length of the intestinal crypt‐villus axis.

## CONFLICT OF INTEREST

The authors declare that there are no conflicts of interest.
