# Supplementary figures and images for: Vasoactive intestinal peptide regulates ileal goblet cell production in mice
Source: Physiol Rep. 2020 Feb 5;8(3):e14363. doi: 10.14814/phy2.14363 (PMC7002535; doi:10.14814/phy2.14363)

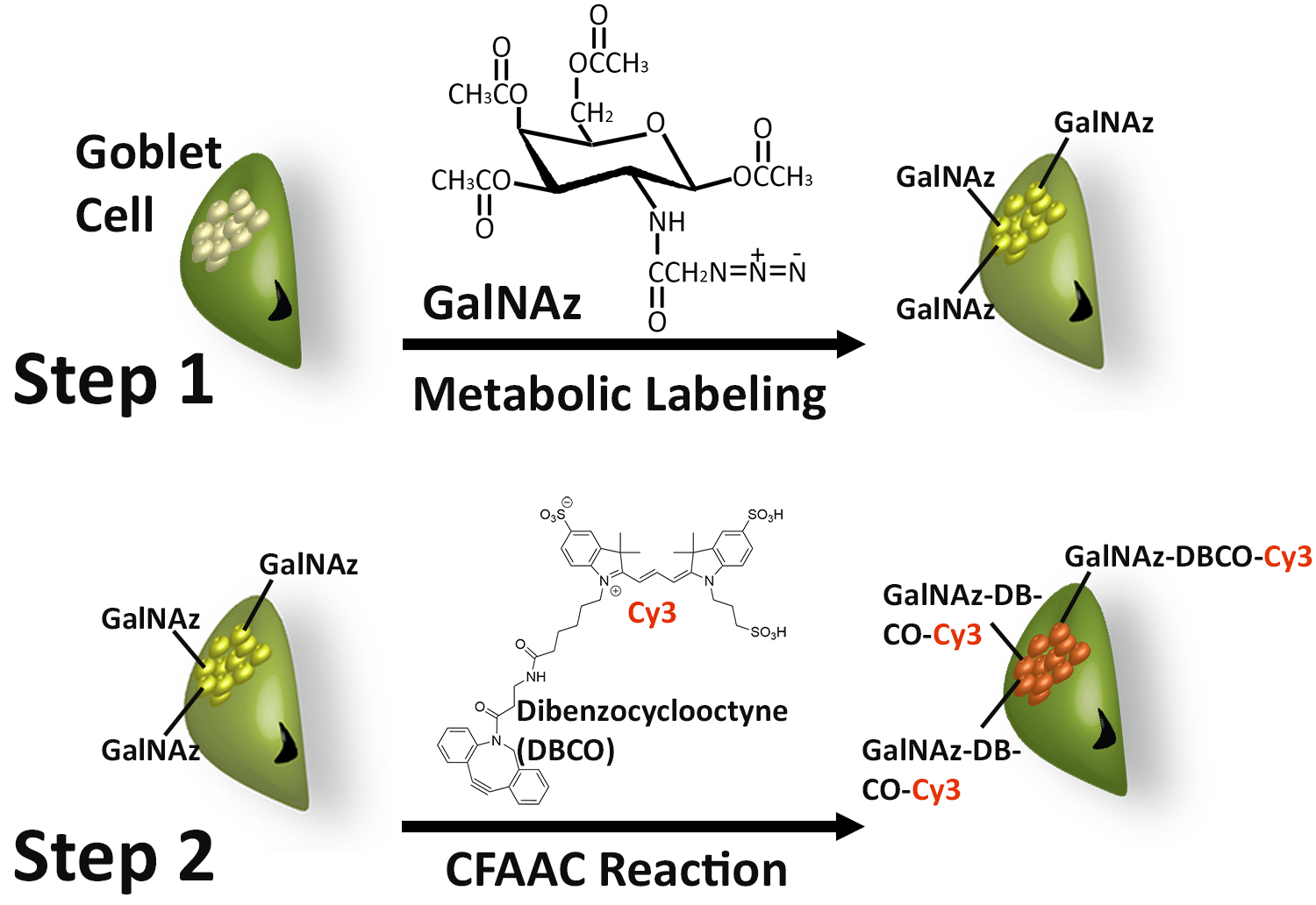

Supplement: Supplementary file 1 [file PHY2-8-e14363-s001.tif]

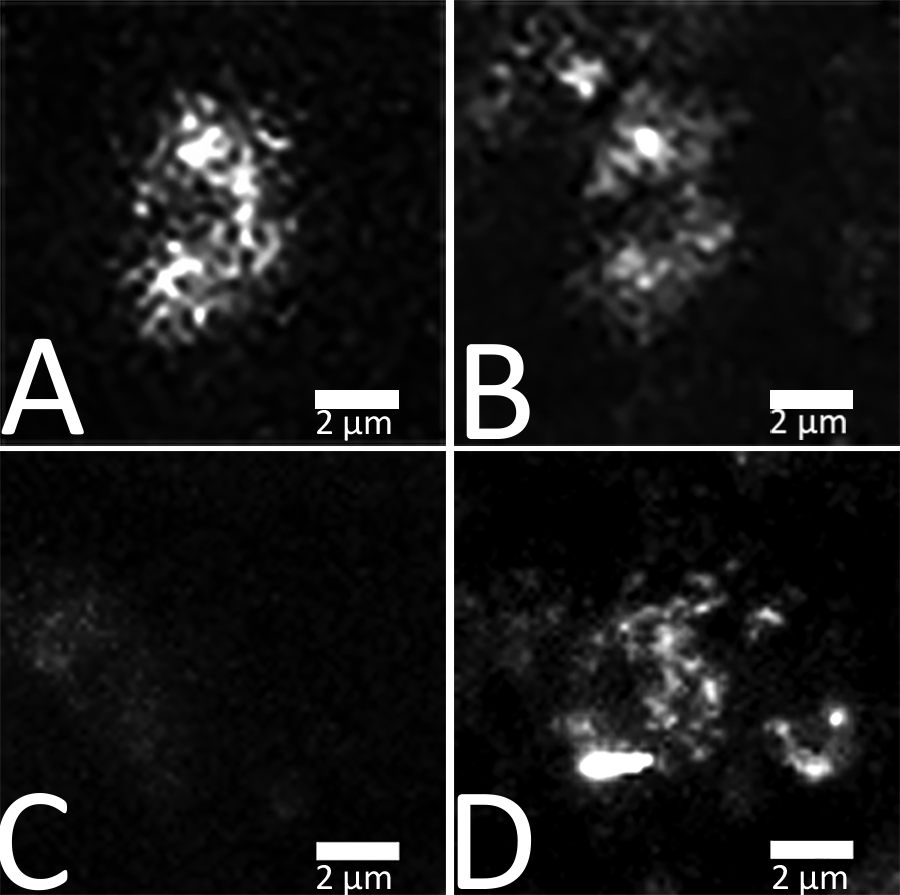

Supplement: Supplementary file 2 [file PHY2-8-e14363-s002.tif]
